# Variation of soil organic carbon and physical properties in relation to land uses in the Yellow River Delta, China

**DOI:** 10.1038/s41598-020-77303-8

**Published:** 2020-11-23

**Authors:** Shuying Jiao, Junran Li, Yongqiang Li, Ziyun Xu, Baishu Kong, Ye Li, Yuwen Shen

**Affiliations:** 1 College of Resources and Environment, National Engineering Laboratory for Efficient Utilization of Soil and Fertilizer Resources, Shandong Agricultural University, No. 61 Daizong Street, Tai’an, 271018 Shandong China; 2 Department of Geosciences, The University of Tulsa, Tulsa, OK 74104 USA; 3 Shandong Academy of Agricultural Sciences, Institute of Agricultural Resources and Environment, No. 202 Gongyebei Road, Jinan, 250100 Shandong China

**Keywords:** Agroecology, Grassland ecology, Wetlands ecology, Agroecology, Grassland ecology, Wetlands ecology, Ecology, Ecology

## Abstract

Soil physical properties and soil organic carbon (SOC) are considered as important factors of soil quality. Arable land, grassland, and forest land coexist in the saline-alkali reclamation area of the Yellow River Delta (YRD), China. Such different land uses strongly influence the services of ecosystem to induce soil degradation and carbon loss. The objective of this study is to evaluate the variation of soil texture, aggregates stability, and soil carbon affected by land uses. For each land use unit, we collected soil samples from five replicated plots from “S” shape soil profiles to the depth of 50 cm (0–5, 5–10, 10–20, 20–30, and 30–50 cm). The results showed that the grassland had the lowest overall sand content of 39.98–59.34% in the top 50 cm soil profile. The content of soil aggregates > 0.25 mm (*R*_0.25_), mean weight diameter and geometric mean diameter were significantly higher in grassland than those of the arable and forest land. *R*_0.25,_ aggregate stability in arable land in the top 30 cm were higher than that of forest land, but lower in the soil profile below 20 cm, likely due to different root distribution and agricultural practices. The carbon management index (CMI) was considered as the most effective indicator of soil quality. The overall SOC content and CMI in arable land were almost the lowest among three land use types. In combination with SOC, CMI and soil physical properties, we argued that alfalfa grassland had the advantage to promote soil quality compared with arable land and forest land. This result shed light on the variations of soil properties influenced by land uses and the importance to conduct proper land use for the long-term sustainability of the saline-alkali reclamation region.

## Introduction

Land uses strongly influence the processes and capacity of ecosystem, causing the change of ecosystem functions and services^1,2^. Understanding ecological consequence of land use conversion is critical for maintaining ecosystem services and conserving biodiversity^3,4^. Conversion of land use from natural ecosystems to agricultural ecosystems may lead to the degradation of most of the ecosystem services, which may pose a direct threat to regional and global substantial environmental developments^5^. One important property of ecosystems that is likely to change with land uses is soil carbon stock, which is linked to carbon dioxide (CO_2_) concentration in the atmosphere^6^ and may have a significant feedback to the global carbon cycle, as the amount of carbon stored in soil is approximately twice more than that in the atmosphere^7,8^. Soil organic carbon (SOC) is the main component of soil carbon stock and plays a critical role in maintaining the services of ecosystems^9^, such as food production, water quality provision, soil fertilization, climate change abatement etc^10,11^. Land use changes have been largely expanded over last several decades due to the increase of population and food demand^12,13^, which may have caused increased CO_2_ emission and intensified greenhouse effect^14^. Hence, variations of SOC as a result of land use changes have caught much attention worldwide as a critically important issue for agricultural management, ecosystem restoration and environmental conservation^5,9^.

Soil physical factors are known to affect SOC. Both aggregate stability and soil particle size are among the most important soil physical properties^15^. Differences in human activities and vegetation types had a strong effect on the changes in soil biological properties^16,17^, and biomass and litter types also affected soil organic matter (SOM)^18^. The SOM is an effective indicator of soil resource condition that reflects functional traits such as soil aggregate stability, water holding capacity, and microbial activity^19^ and had close relationship with aggregate stability and soil erodibility^20^. Therefore, the loss of soil carbon with disturbed cultivation was often linked to the deterioration of soil physical properties. Conversely, soil aggregation structure had a great extent to affect soil biological and physic-chemical processes^21^, and was also closely related to SOM content. Thus, changes of soil aggregation may play an important role in SOC stock and soil quality under cultivation^22^. Soil bulk density (BD) and porosity are functions of SOM, soil particle size and aggregate stability, and soil particle density. Decrease of SOM would cause the increase of BD and the decrease of porosity, consequently reducing soil infiltration, and water and air storage capacities^20^.

Stocks of SOC generally decreased after land use conversion from grassland or forests to arable land due to the decreased carbon input and physical protection of SOM^23^, and the increased aboveground carbon output in arable land. Tillage can break soil aggregates through the action of soil disturbance and make SOM within aggregates exposed to microbial decomposition^24^. According to the conceptual models of soil aggregation, aggregates of different sizes have different strength of SOM protection^25^. So agricultural practices within land uses may be the important reason to alter SOC stocks^26^, such as reduced tillage, no-till with straw retention and conventional tillage. The SOC size-fractionation was a sensitive indictor and was used to assessing short-term SOC changes induced by land uses^22,27^. The coarse (2–0.05 mm) organic carbon represented the recently residues derived from litters and dead roots^28^. Specifically, the identification of more sensitive SOC fractions would help to detect changes in SOC pools with different land uses^29^. Among different SOC pools, those associated with sand fraction and particulate organic matter intimately showed early alterations in SOC resulting from land uses^23,30^. Also some studies reported that variation in SOM content through different land uses would lead to a large modification in soil BD, porosity, infiltration rate, aggregate stability and ultimately cycling^23^, and land use changes played a critical role in soil moisture^31^.

This study site is located at an area with a fragile wetland ecosystem under saline-alkali stress in the Yellow River Delta (YRD) of China. Frequent land use conversion occurred in this area due to anthropogenic activities and secondary salinization, which leads to serious soil degradation and threats the sustainability of the ecosystem. Charactering carbon changes and soil properties among different land uses is essential to assess the impacts of land uses on local ecosystem. Some studies have examined the impacts of different land uses on SOC or soil physicochemical properties in the YRD, but few studies have provided a comprehensive (e.g., deep in the soil profile of 0–50 cm, high density of sampling etc.) understanding of soil physical properties, soil carbon and their relationship among different land uses. Understanding these relationships in the saline-alkali reclamation region may be of particular importance for developing proper management practices for sustainable production. The objectives of this study were: (1) to assess the effect of different land uses on soil particle size and aggregate stability, (2) to determine the effect of land uses on soil carbon and (3) to provide insights into the coupling relationships between soil physical properties and soil carbon influenced by different land uses. These results are expected to help improve the understanding to conduct proper land uses and agricultural management strategies for the long-term sustainability of the saline-alkali reclamation region.

## Results

### Soil physical properties for different land uses

Soil water content varied significantly among different land uses (*P* < 0.05), and it was lower in the top layers than the deeper layers and followed the order: arable land > grassland > forest land (Table 1). Soil bulk density (BD) and soil porosity (P_t_) among the land uses varied slightly, and significant differences were only detected in the layers of 10–20 cm and 20–30 cm (Table 1).

**Table 1:** Soil water content (SWC), soil bulk density (BD) and total soil porosity (P_t_) among different land uses (mean ± SD).

Soil depths	Land uses	BD (g cm^−3^)	SWC (%)	Pt (%)
0–5 cm	AL	1.33 ± 0.11a	13.80 ± 1.42a	49.94 ± 4.13a
	GL	1.28 ± 0.04a	8.84 ± 1.12b	51.84 ± 1.61a
	FL	1.32 ± 0.07a	3.72 ± 1.57c	50.12 ± 2.68a
5–10 cm	AL	1.30 ± 0.08a	16.34 ± 0.43a	50.92 ± 3.11a
	GL	1.37 ± 0.05a	11.12 ± 0.20b	48.28 ± 2.11a
	FL	1.34 ± 0.06a	5.79 ± 1.52c	49.46 ± 1.99a
10–20 cm	AL	1.25 ± 0.10b	18.13 ± 0.32a	52.82 ± 3.71a
	GL	1.50 ± 0.02a	13.09 ± 0.45b	43.44 ± 0.54b
	FL	1.28 ± 0.01b	5.69 ± 0.66c	51.77 ± 0.21a
20–30 cm	AL	1.39 ± 0.04a	20.56 ± 1.02a	47.40 ± 1.32b
	GL	1.38 ± 0.02a	15.15 ± 0.10b	47.75 ± 0.72b
	FL	1.29 ± 0.04b	8.24 ± 0.42c	51.10 ± 1.24a
30–50 cm	AL	1.34 ± 0.06a	22.20 ± 0.38a	49.48 ± 2.16a
	GL	1.36 ± 0.07a	18.56 ± 0.38a	48.66 ± 2.65a
	FL	1.33 ± 0.02a	12.20 ± 4.39b	49.90 ± 0.67a

Different letters at the same soil depth indicate significant difference (n = 5, *p* < 0.05) among different land uses by one-way ANOVA.

**Figure 1:**
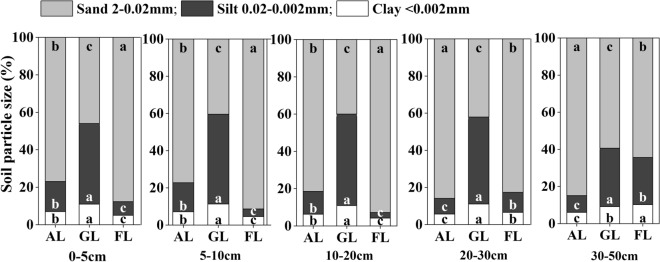
Soil particle size among different land uses at 0-50 cm depth. Different letters with the same particle size at the same soil depth indicate significant difference (n = 5, *p* < 0.05) among different land uses by one-way ANOVA.

Soil particle size among different land uses varied significantly. Soil texture was mainly composed of silt and sand, and the clay only occupied for less than 12% among three land uses in the YRD, and sand was dominant soil particle in arable land and forest land, accounting for nearly 80%. Silt and clay contents were generally the highest in grassland among different land uses in each layer of the top 50 cm soil profile (Fig. 1).

Land uses had strong effect on soil aggregates. The aggregate content at *R*_*0.25*_ had significant difference among different land uses (Table 2), and this aggregate content was generally the highest in grassland among different land uses in the entire top 50 cm soil profile. Stability of soil aggregates, as measured by mean weight diameter (MWD) and geometric mean diameter (GMD), followed the order of grassland > arable land > forest land (Table 3).

**Table 2:** Effect of land uses on soil dry-stable aggregates (%) at different depth (mean ± SD).

Soil depths	Land uses	> 5 mm	5–2 mm	2–1 mm	0.5–1 mm	0.5–0.25 mm	*R*_*0.25*_
0–5 cm	AL	14.01 ± 1.68a	14.18 ± 4.36a	6.54 ± 1.30b	8.03 ± 1.48b	5.64 ± 1.64b	48.41 ± 9.27b
	GL	10.57 ± 0.43b	16.06 ± 2.80a	14.30 ± 1.76a	24.80 ± 3.24a	14.15 ± 2.46a	79.89 ± 4.59a
	FL	1.98 ± 1.12c	3.03 ± 0.87b	1.70 ± 0.56c	2.14 ± 0.62c	2.37 ± 0.95c	11.22 ± 3.50c
5–10 cm	AL	16.15 ± 2.57b	14.86 ± 1.16b	7.86 ± 1.50b	8.60 ± 1.29b	5.58 ± 0.51b	53.04 ± 4.74b
	GL	23.67 ± 4.84a	23.72 ± 1.91a	12.60 ± 0.81a	14.51 ± 2.14a	8.41 ± 2.15a	82.93 ± 3.92a
	FL	4.44 ± 3.65c	4.98 ± 3.63c	1.55 ± 0.87c	1.31 ± 0.69c	1.30 ± 0.44c	13.57 ± 9.06c
10–20 cm	AL	23.04 ± 3.80b	13.21 ± 2.69b	8.12 ± 2.76b	8.64 ± 1.84b	5.17 ± 0.74b	58.17 ± 7.01b
	GL	37.49 ± 3.75a	25.65 ± 2.10a	10.98 ± 0.53a	11.78 ± 1.55a	6.15 ± 0.66a	92.04 ± 1.49a
	FL	2.59 ± 0.57c	3.35 ± 0.57c	1.28 ± 0.16c	1.35 ± 0.15c	1.46 ± 0.53c	10.03 ± 1.07c
20–30 cm	AL	7.06 ± 2.79c	19.48 ± 11.30a	5.60 ± 1.24b	7.09 ± 2.70b	3.86 ± 1.29b	43.07 ± 16.94b
	GL	39.45 ± 4.08a	25.89 ± 1.39a	9.77 ± 2.81a	10.76 ± 1.14a	5.38 ± 0.75a	91.25 ± 3.77a
	FL	13.68 ± 0.98b	8.58 ± 1.43b	4.53 ± 2.50b	3.62 ± 1.78c	2.37 ± 0.95c	32.78 ± 6.21b
30–50 cm	AL	9.97 ± 0.43c	12.45 ± 4.83c	6.04 ± 0.22b	4.99 ± 2.04b	3.00 ± 1.58a	36.45 ± 8.49c
	GL	44.76 ± 4.87a	31.28 ± 4.36a	10.62 ± 1.52a	9.13 ± 0.98a	3.67 ± 0.49a	99.47 ± 6.88a
	FL	23.50 ± 3.97b	24.63 ± 1.41b	9.49 ± 1.35a	7.03 ± 1.54ab	3.81 ± 0.59a	68.46 ± 3.85b

Different letters with the same soil depth indicate significant difference (n = 5, *p* < 0.05) between different land uses by one-way ANOVA. *R*_*0.25*_ is aggregates of diameter > 0.25 mm.

**Table 3:** MWD, GMD values and PAD_0.25_ of soil dry stable aggregates for different land uses (mean ± SD).

Parameters	Land uses	Soil depth (cm)					Average
		0–5	5–10	10–20	20–30	30–50	
MWD (mm)	AL	1.50 ± 0.24a	1.65 ± 0.12b	1.93 ± 0.19b	1.33 ± 0.46b	1.23 ± 0.18c	1.53 ± 0.19b
	GL	1.59 ± 0.10a	2.39 ± 0.27a	3.07 ± 0.14a	3.15 ± 0.17a	3.58 ± 0.32a	2.75 ± 0.04a
	FL	0.48 ± 0.08b	0.65 ± 0.30c	0.51 ± 0.05c	1.26 ± 0.09b	2.33 ± 0.18b	1.04 ± 0.11c
GMD (mm)	AL	0.71 ± 0.15b	0.78 ± 0.07b	0.92 ± 0.14b	0.66 ± 0.23b	0.56 ± 0.09c	0.73 ± 0.12b
	GL	0.95 ± 0.08a	1.46 ± 0.24a	2.15 ± 0.16a	2.22 ± 0.24a	3.01 ± 0.65a	1.96 ± 0.08a
	FL	0.31 ± 0.02c	0.35 ± 0.08c	0.31 ± 0.01c	0.54 ± 0.05b	1.27 ± 0.14b	0.55 ± 0.05c
PAD_0.25_	AL	97.33 ± 0.40a	97.69 ± 0.40c	98.20 ± 0.55a	98.88 ± 0.15a	99.66 ± 0.36a	98.35 ± 0.09a
	GL	95.67 ± 1.22b	98.41 ± 0.35b	98.25 ± 0.53a	96.26 ± 3.02b	98.07 ± 0.98b	97.33 ± 0.65b
	FL	97.21 ± 0.45a	99.14 ± 0.50a	98.15 ± 0.41a	99.67 ± 0.04a	98.83 ± 0.96ab	98.60 ± 0.17a

Different letters with the same soil depth indicate significant difference (n = 5, *p* < 0.05) among different land uses by one-way ANOVA.

*MWD* the mean weight diameter of dry stable aggregates, *GMD* the geometric mean diameter of dry stable aggregates, *PAD*_*0.25*_ the > 0.25 mm percentage of aggregate disruption.

### Soil organic carbon fractions for different land uses

**Figure 2:**
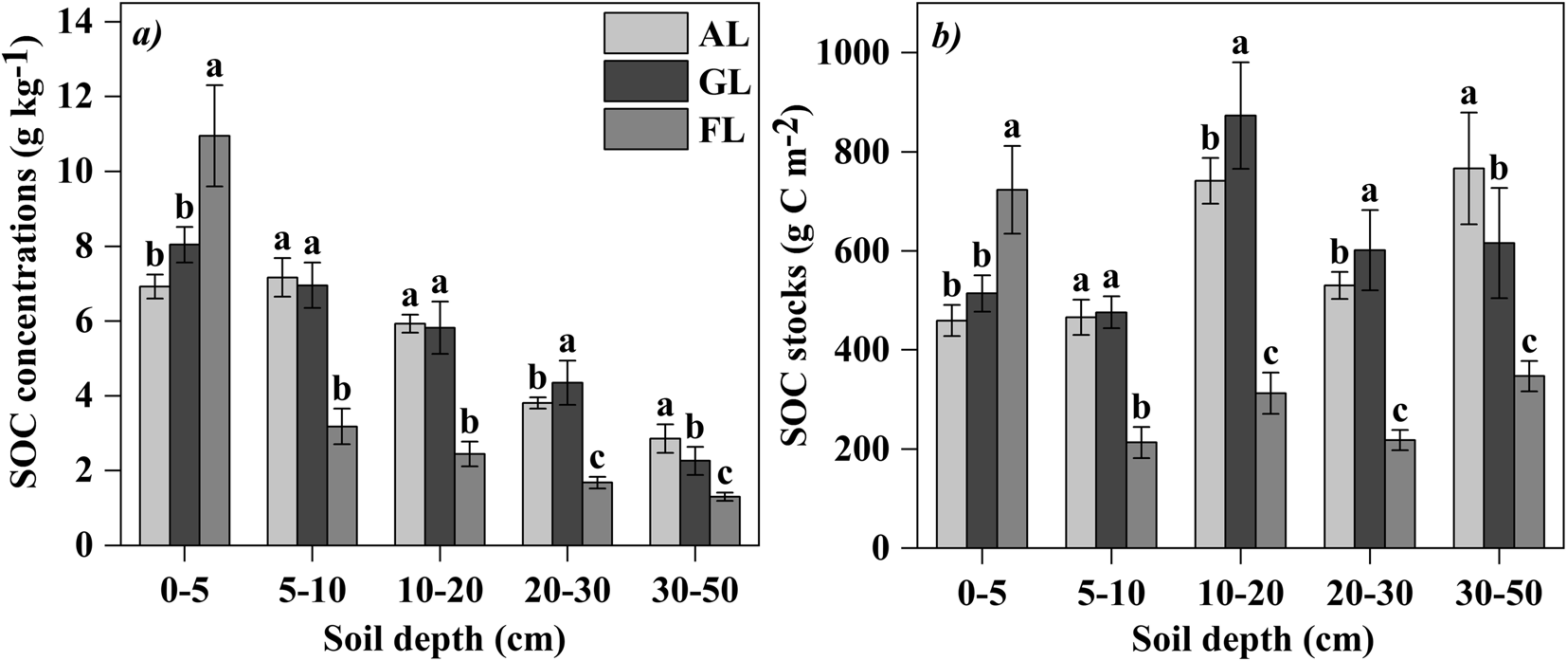
(**a**) Soil organic carbon (SOC) concentration and (**b**) SOC stock (mean ± SD) with different land uses. Different letters at the same soil depth indicate significant difference (n = 5, *p* < 0.05) among different land uses by one-way ANOVA.

The concentration of SOC in forest land was significantly higher than arable land and grassland in the top 5 cm soil layer (Fig. 2a). The overall SOC stock showed similar patterns with SOC concentration among different land uses, except for the grassland in 10–30 cm soil profile, which was significant higher than arable land and forest land (Fig. 2b).

**Figure 3:**
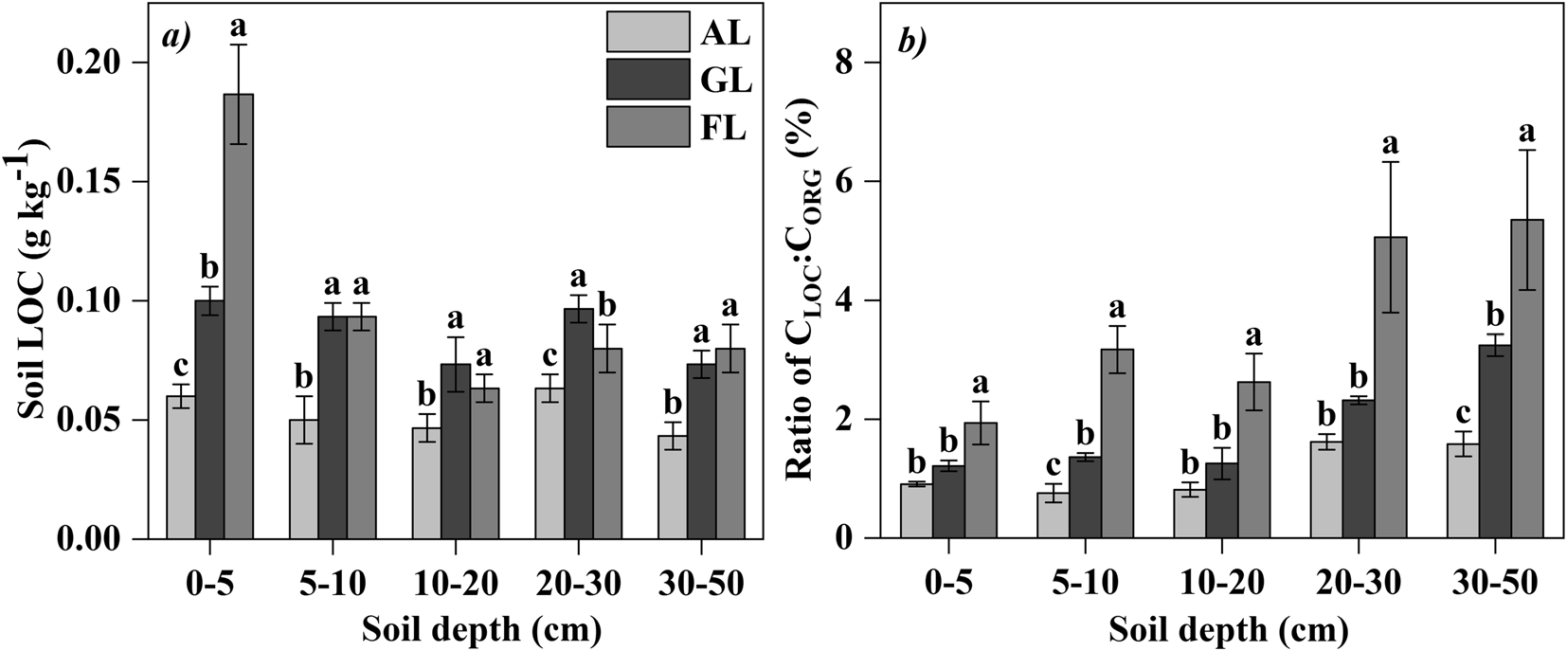
(**a**) Soil labile carbon (LOC) concentration and (**b**) the C_LOC_ to C_ORG_ ratios (mean ± SD) with different land uses. Different letters at the same soil depth indicate significant difference (n = 5, *p* < 0.05) among different land uses by one-way ANOVA.

The concentration of soil labile carbon (LOC) in arable land was significant lower than both grassland and forest land in the top 50 cm soil profile (Fig. 3a). The C_LOC_ to C_ORG_ ratio followed the order of forest land > grassland > arable land (Fig. 3b).

**Figure 4:**
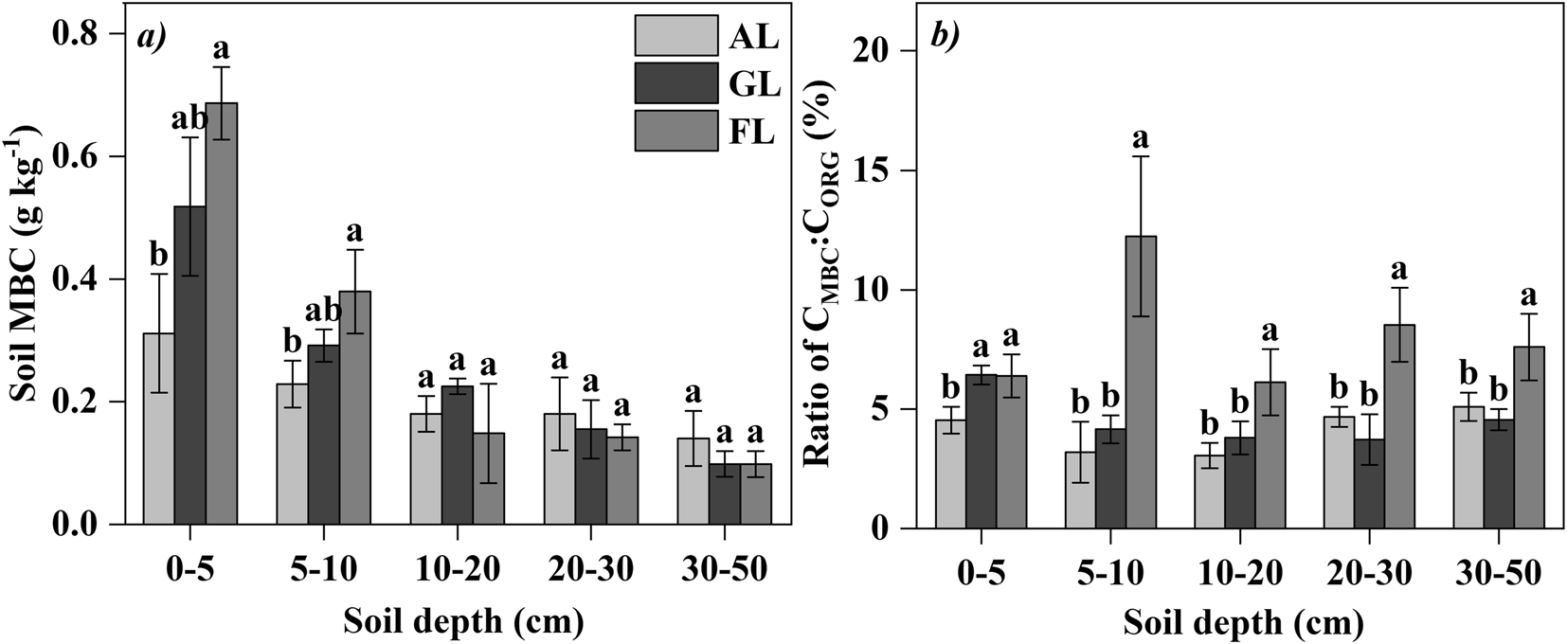
(**a**) Soil microbial biomass carbon (MBC) concentration and (**b**) the C_MBC_ to C_ORG_ ratios (mean ± SD) with different land uses. Different letters at the same soil depth indicate significant difference (n = 5, *p* < 0.05) among different land uses by one-way ANOVA.

The concentration of soil microbial biomass carbon (MBC) decreased with the increase of soil depth among different land uses and followed the order of forest land > grassland > arable land (Fig. 4a). The C_MBC_ to C_ORG_ ratio was significantly higher in forest land than in arable land and grassland at soil profile below 5 cm (*p* < 0.05) (Fig. 4b).

We calculated the carbon management index (CMI) for the soil of forest land and used it as the reference soil. The relationship between NL and CPI and between L and LI had the same patterns of variation (Table 4). The CMI showed significant difference among different land uses, and changed according to the patterns of the LOC concentration. The CMI in arable land in the top 50 cm soil profile was the lowest, and decreased with the increase of soil depth. The CMI in grassland was more than 100 in the depths of 10–20 cm and 20–30 cm, suggesting that the CMI of grassland was higher than forest land (reference soil).

**Table 4:** Soil carbon management index (CMI) with different land uses (mean ± SD).

Soil depths	LAND uses	NL (g kg^-1^)	L	LI	CPI	CMI
0–5 cm	AL	6.86 ± 0.24b	0.01 ± 0.00b	0.46 ± 0.02c	0.70 ± 0.02c	32.63 ± 0.97c
	GL	7.99 ± 0.51ab	0.01 ± 0.00b	0.62 ± 0.05b	0.82 ± 0.05b	50.96 ± 0.81b
	FL	9.66 ± 1.52a	0.02 ± 0.00a	1.00 ± 0.00a	1.00 ± 0.00a	100.00 ± 0.00a
5–10 cm	AL	7.10 ± 0.29a	0.01 ± 0.00c	0.23 ± 0.05c	2.37 ± 0.09a	54.72 ± 9.50b
	GL	6.82 ± 0.17a	0.01 ± 0.00b	0.42 ± 0.02b	2.29 ± 0.06a	96.37 ± 6.47a
	FL	2.93 ± 0.36b	0.03 ± 0.00a	1.00 ± 0.00a	1.00 ± 0.00b	100.00 ± 0.00a
10–20 cm	AL	6.10 ± 0.26a	0.01 ± 0.00b	0.3 ± 0.05c	2.56 ± 0.11a	77.39 ± 8.74b
	GL	5.64 ± 0.35a	0.01 ± 0.00b	0.47 ± 0.1b	2.37 ± 0.14a	110.78 ± 18.21a
	FL	2.34 ± 0.45b	0.03 ± 0.01a	1.00 ± 0.00a	1.00 ± 0.00b	100.00 ± 0.00ab
20–30 cm	AL	3.75 ± 0.03a	0.02 ± 0.00b	0.31 ± 0.03c	2.31 ± 0.02b	71.05 ± 6.54c
	GL	4.15 ± 0.31a	0.02 ± 0.00b	0.44 ± 0.01b	2.58 ± 0.19a	114.35 ± 5.64a
	FL	1.57 ± 0.54b	0.05 ± 0.01a	1.00 ± 0.00a	1.00 ± 0.00c	100.00 ± 0.00b
30–50 cm	AL	2.83 ± 0.04a	0.02 ± 0.00c	0.28 ± 0.04c	1.84 ± 0.02a	52.08 ± 6.64c
	GL	2.20 ± 0.14a	0.03 ± 0.00b	0.59 ± 0.03b	1.45 ± 0.09b	86.11 ± 8.08b
	FL	1.48 ± 0.53b	0.06 ± 0.01a	1.00 ± 0.00a	1.00 ± 0.00c	100.00 ± 0.00a

Different letters with the same soil depth indicate significant difference (n = 5, p < 0.05) between different land uses by one-way ANOVA.

*NL* non-labile carbon concentration (g kg^−1^), *L* Carbon pool lability, *LI* lability index, *CPI* Carbon pool index.

### Relationship between soil carbon and physical properties

Pearson’s correlation revealed that the CPI was significantly (*P* < 0.01) and positively correlated with the SWC, *R*_*0.25*_, MWD, GMD, BD and silt content (Table 5). The C_MBC_ to C_ORG_ ratio was strongly positively correlated with sand content, but negatively correlated with the contents of clay, silt and *R*_*0.25*_, MWD and GMD. A similar, negative correlation was also found between MBC and *R*_*0.25*_, MWD and GMD. Finally, we found that SWC was negatively correlated with LOC, MBC, C_MBC_ to C_ORG_ ratio and LI.

**Table 5:** Pearson’s correlation between the characteristics of soil carbon and soil physical properties.

Factors	SWC (%)	BD (g cm^−3^)	P_t_ (%)	Clay (%)	Silt (%)	Sand (%)	*R*_*0.25*_	MWD	GMD
SOC	− 0.193	− 0.028	0.039	0.120	0.214	− 0.203	0.050	− 0.125	− 0.136
LOC	− 0.639**	− 0.014	0.022	0.027	0.093	− 0.086	− 0.177	− 0.202	− 0.078
MBC	− 0.508**	− 0.243	0.246	− 0.223	− 0.147	0.155	− 0.366*	− 0.450**	− 0.378*
LOC:SOC	− 0.260	− 0.072	0.069	− 0.007	− 0.143	0.125	− 0.124	0.013	0.040
MBC:SOC	− 0.391**	− 0.207	0.204	− 0.356*	− 0.360*	0.360*	− 0.462**	− 0.413**	− 0.371*
NLC	− 0.186	− 0.027	0.038	0.121	0.215	− 0.203	0.053	− 0.123	− 0.136
L	− 0.256	− 0.072	0.070	− 0.004	− 0.142	0.124	− 0.122	0.014	0.037
LI	− 0.801**	− 0.215	0.216	− 0.253	− 0.288	0.283	− 0.469**	− 0.402**	− 0.281
CPI	0.599**	0.333*	− 0.334*	0.266	0.338*	− 0.329*	0.442**	0.483**	0.390**
CMI	− 0.408**	0.213	− 0.215	0.142	0.209	− 0.203	0.050	0.208	0.290

*Correlation significant at the 0.05 level (two-tailed).

**Correlation significant at the 0.01 level (two-tailed).

## Discussion

Soil physical properties are critical to soil quality in aspects of root growth, infiltration, water and nutrient holding capacity^32^. Land uses and vegetation types can significantly influence soil physical properties, particularly soil aggregates distribution^33,34^. In this study, the variation of SWC, BD and porosity occurred among three land uses, the higher SWC observed in arable land was likely related to the fact that farmland undergone the artificial irrigation. The lower SWC in forest land than that in alfalfa grassland was probably related to the lower surface cover and the characteristic of roots in forest land, which both lead to greater transpiration^35,36^. This result agreed with the study of Zhang et al. (2016) that SWC at the grass stage was a significantly higher than that at the forest stage^32^. The high soil BD of the arable land was likely the result of combined influence of the ploughing in tillage layer, roots distribution and decreased SOC and soil aggregation, as a result of repeated events of sowing and harvesting^20,37^.

Soil particles and soil aggregate are the important physical properties for the process of soil physiochemical and biological properties, and soil particle size distribution is the fundamental physical factor affecting aggregate stability^38^. Results of our study indicated that sand is the primary soil particle among three land uses in the YRD, and the lowest sand content of 39.98–59.34% occurred in the grassland in the top 50 cm soil profile (Fig. 1). This result may be explained by different effects of soil erosion control under different land uses^39^. The vegetation types, coverage, and root system condition among different land uses are correlated to soil particle composition; higher root growth and litter input can improve soil physiochemical and biological properties, accelerate the formation of humus, reduce the surface wind erosion and facilitate the fixation of fine sand particles. In the grassland, high vegetation coverage and root activity can prevent soil erosion from rain splash therefore the loss of fine soil grains, because root activity of plant could greatly affect the distribution of soil particle size in newly formed wetlands in the YRD^34^. The soil aggregates were mainly non water-stable aggregates, and the number of water-stable aggregates was very small (Tables S1), which may be related with the special saline-alkali environment and local soil texture due to its new and fast formation of alluvial plain. To a certain extent, the situation of water-stable aggregates affects soil aeration and erosion resistance, and its small portion indicates the poor soil fertility and stability in this region. Averaged across three land uses, the contents of silt, clay and soil dry-stable aggregates (*R*_*0.25*_) in grassland was highest among all three land uses studies, which is in agree with a previous study by Liu et al.^40^. It should be noted that tillage and harvesting practices in arable land and low vegetation coverage in forest land may promote soil erosion and cause the loss of silt and clay contents and the decrease of soil aggregate stability in topsoil^41,42^. The alfalfa plants generally have a well-developed root system, which produces more organic matter as the roots decompose. High content of soil organic matter will produce more soil aggregates and improve the soil structure^20,43^.

Soil acts as either a carbon source or a carbon sink, and land uses can change the function of source and sink^32,44^. For the top soil (0–5 cm), our study found that SOC content and stock in forest land were the highest, which was resulted from the input of litter on the surface soil. While the arable land had less litter, frequent disturbance and strong soil respiration in the surface, which accelerated the consumption of SOC in the top soil^45^. But at the deeper section of the soil profile (5–50 cm), SOC content and stock in arable land and grassland were higher than that in forest land due to continuous root production and decomposition. This result was probably related to the rich root system of arable land and grassland concentrated in the deeper soil profile^18,46^, while lower root production and poor soil permeability in forest land, because different vegetation type can regulate the distribution of SOC through plant growth and root distribution, and the lack of oxygen soil has a fundamental restriction on microbial decomposition^47^. Some studies confirmed that vegetation restoration and belowground biomass had a close relationship with SOC^48^, and played a critical role in improving SOC stock in a degraded salt land^47^. Chen et al. confirmed that soil carbon accumulation was strongly driven by the establishment of vegetation^49^. As a salt-tolerate plant, alfalfa has high biomass and root activity, which is the main reason why artificial alfalfa grassland has high carbon content and stock compared to arable land and forest land in the saline-alkali reclamation region. Xiao et al.^50^ showed that conversion of natural system to other land uses decreased MBC, and the content of the LOC in TOC indicates soil quality^51,52^. As CMI is a good indicator of soil carbon quality^53^, we argued that alfalfa grassland, which has the highest CMI values, seems to provide better options for soil carbon management and soil quality.

**Figure 5:**
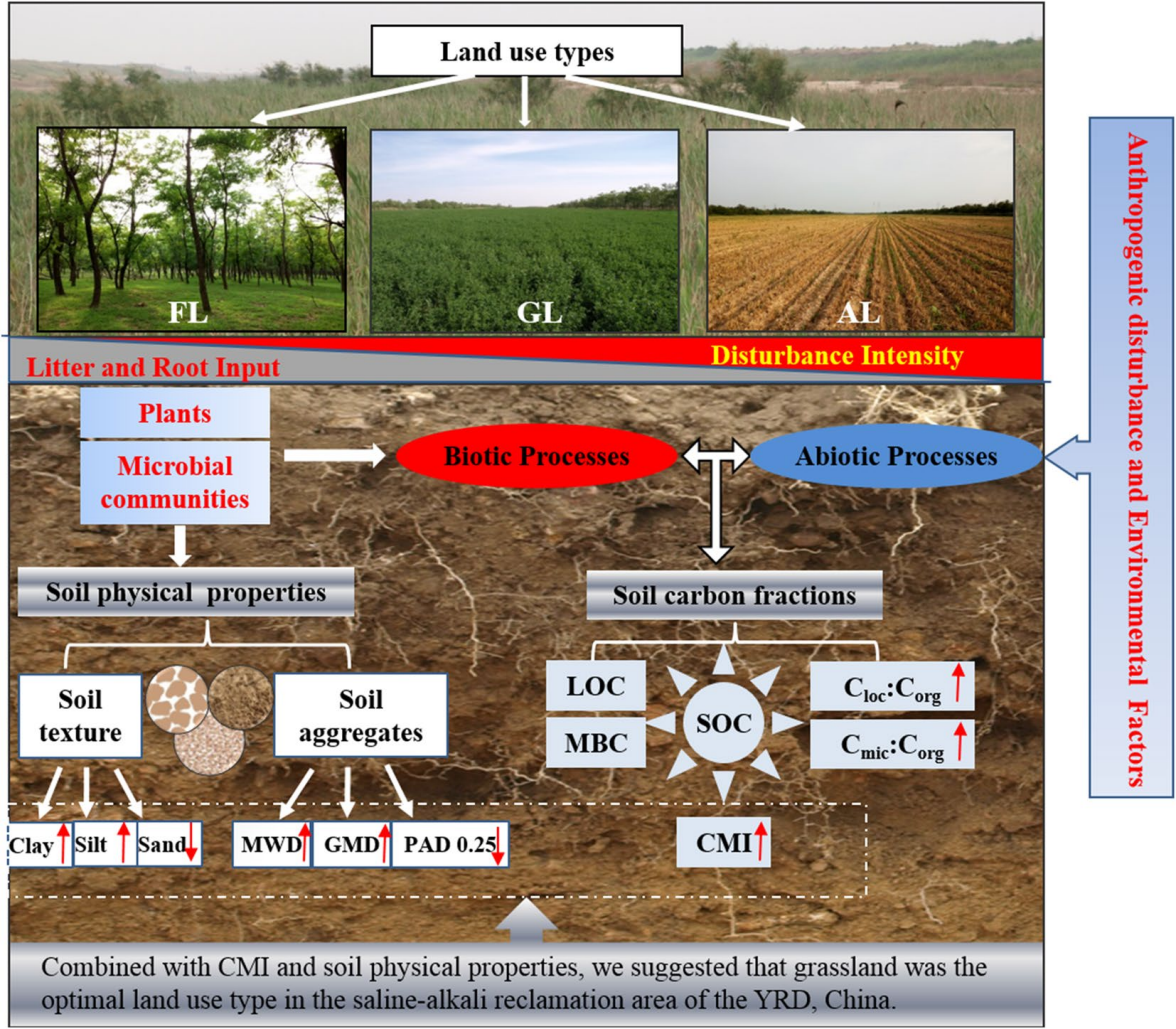
Relationship between soil physical properties and soil organic carbon with different land uses. The photographs in Fig. 5 were taken from three land uses (FL, GL, AL) in the study area by the author ‘Shuying Jiao’.

Soil organic carbon, particularly active component of organic carbon, has been reported to act as important binding agents for soil aggregates and their stability^54,55^, and this assertation was demonstrated by the strong correlation between CPI and soil physical properties in our study. Moreover, we found that CPI were particularly sensitive to soil water content (SWC) and aggregate stability. According to Zhao et al., higher SWC could reduce the impact of soil salinity on soil carbon stock due to the variations of salt concentrations and O_2_ diffusion of soil layers, because high salinity could influence solubility of SOM, inhibit microbial processes and the final soil carbon stock^47^, which could explain higher carbon content and stock in arable land and grassland than those in forest land in the deeper soil profile. Increased SOC could improve aggregate stability indirectly through increasing energy and nutrient availability for soil microbes^53^. Moreover, some studies found that the aggregate stability was related to SOM composition and had good correlations between carbohydrate content and soil aggregate stability^40^. Specifically, the occurrence of SOC and aggregate stability in arable land and grassland were higher than that in forestland in this region studies, but lower active component of organic carbon in arable land which resulted in lower carbon management. In brief, land use types had changed the vegetation types with different disturbance intensities, litter and roots inputs. The soil physical properties changed through biotic process regulated by plants and soil microbial communities; the inputs and accumulation of organic matter resulted from complex interactions between biotic processes and abiotic processes driven by anthropogenic disturbance and environmental factors (Fig. 5). Under different vegetation types, the soil texture and soil aggregate stability was improved; SOC and active carbon increased, followed by the increase of CMI in grassland. Therefore, combining soil physical properties and soil carbon index with land uses in comprehensive consideration, we believed that alfalfa grassland is the best land use type to improve soil physical properties and the quality of soil carbon in the YRD, which experienced frequent secondary salinization in the past decades. As a result, the proper land use, or the conversion of forest and grassland into arable land should be of concern in the context of soil quality and environmental degradation. In view of the arable land, to conduct proper agricultural practices, such as less impact of land tillage practices, may be a better option to improve soil quality for the long-term production and sustainability of the saline-alkali reclamation region.

## Conclusions

Soil physical properties play an important role in the formation and transformation of soil carbon during the process of land use and land cover change. The study showed strong changes in soil physical properties and soil carbon among the arable land, grassland and forest land and these changes were not uniform along the soil profile to the depth of 50 cm. Overall, we found that alfalfa grassland had effectively improved soil physical properties and soil carbon, and the soil layer of 20–30 cm may be the turning point for soil physical properties change between the arable land and forest land. Land management practices, such as plowing and harvesting, had strong impact on the SOC, suggested by higher SOC in the arable land compared to forest land except 0–5 cm soil layer. Additionally, CMI of arable land was the lowest relative to grassland and forest land. Therefore, the long-term conventional cultivation of arable land is not favorable to soil carbon management and soil quality improvement, and more attention should be paid to improve the soil quality and ecosystem sustainability in the saline-alkali reclamation region.

## Materials and methods

### Study site

**Figure 6:**
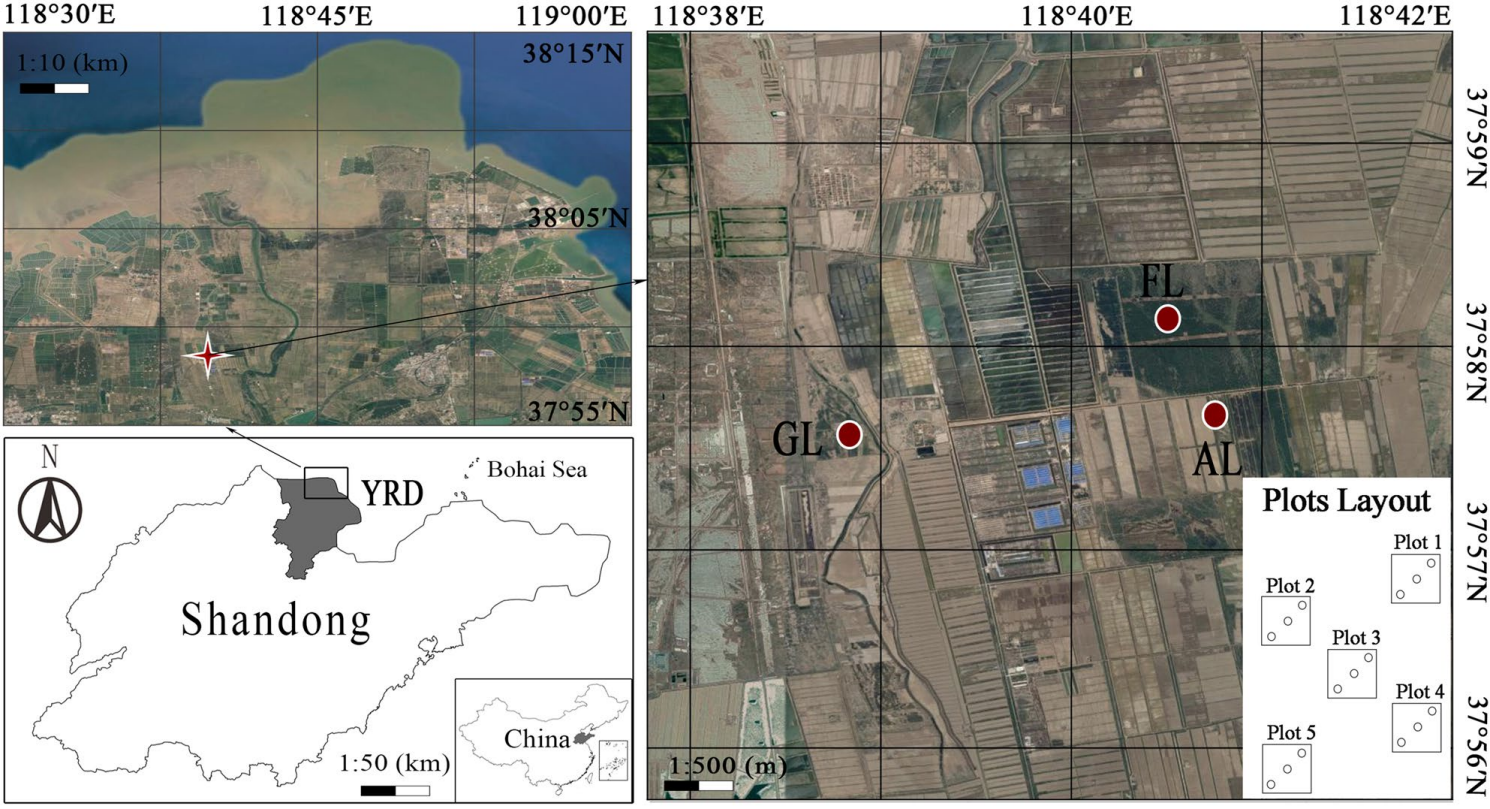
The location of the study area in the Yellow River Delta of Shandong Province and the layout of the study plots (not to scale); this map was created using the software of Photoshop CS6 and Bigemap 14.1 https://www.bigemap.com/.

The study sites are located in the Hekou district of Dongying city, in the Yellow River Delta (YRD), Shandong Province, China (37°54′10.19″ N, 118°31′13.83″ E Fig. 6). It is a typical alluvial plain of the Yellow River and belongs to the semi-humid monsoon climate zone with warm temperate. Mean annual precipitation is about 692 mm occurring mainly during June, July and August, and mean annual temperature is 13.2 °C with seasonal variation. The soils are mainly of Calcaric Fluvisols (moisture soil) and Gleyic Solonchaks (coastal saline moisture soil) according to FAO^56^. The mixed forest is dominated by a *Robinia pseudoacacia* L. and an adjacent crop land situated side by side for the study. The cultivation vegetation after reclamation consists of wheat–maize and purple alfalfa predominantly, the arable land and artificial grassland sometimes were converted to each other due to the serious secondary salinization.

### Experimental design

Three typical land use units (annual arable land, artificial grassland and artificial forestland) were selected according to the main land use types in the study area, and were investigated in detail for the history and current situation of land cultivation. The annual arable land was ploughed, fertilized, irrigated and planted with crop every year since reclamation of the 1950s, and rotationally planted with winter wheat (*Triticum aestivum* L.) and summer maize (*Zea may* L.) under conventional tillage for more than ten years. Winter wheat was sown in early October and harvested in early June next year, and summer maize was sown in mid-June and harvested in late September. The artificial grassland was transformed from the previous arable land in 2011, and then continuously planted with purple alfalfa (*Medicago sativa* L.) for five years. The alfalfa was harvested for four times each year as a source of livestock fodder. For the alfalfa field, base fertilizer was applied at sowing and no other fertilizer was applied during five-year of growth. The forestland is the result of artificial afforestation occurred in the 1960s on the saline-alkali land dominated with *Robinia pseudoacacia* L. without reclamation. The three types of land use have similar physiographic conditions and slope gradients, which belongs to the same region and has the same parent materials. The crop and plantation of the study sites are as follows: (1) annual arable land (AL): wheat–maize rotation plantation; (2) artificial grassland (GL): alfalfa pasture; (3) Forest land (FL): *Robinia pseudoacacia* L. forest.

### Field work

A field survey was conducted in late September in 2016 (after the summer maize was harvested). In this study, we determined the sampling areas according to the size of the communities^30^, selecting five 2 m × 2 m plots from representative terrain in the herbaceous communities of the arable land and artificial grassland, five 5 m × 5 m plots in the forest land. The sampling plots were distributed according to a “S” shape in each land use unit, and 100 m apart between sampling plots. At each sampling plot, three soil sampling sites were located at the two diagonal corners and the center of the plot. Soils at each sampling site were collected from soil profiles to 50 cm depth (0–5, 5–10, 10–20, 20–30, and 30–50 cm) by using a drill. A composite soil sample at certain soil depth was obtained by mixed all these samples at each plot. The composite soil samples for soil water content (SWC) were stored in sealed aluminum cases to prevent potential moisture loss. Soil samples for SOC fractionation, particle sizes were stored in zip-top plastic bags. The undisturbed soil samples for aggregate analysis were wrapped up with paper to avoid destroying the aggregates. Soil bulk density (BD) was measured at the intermediate position of each soil layer using a cutting ring with inner diameter of 5.0 cm, and volume of 100 cm^3^. Overall, 75 composite soil samples were collected, representing three land uses, five depths and five replicates. The fresh soil samples were immediately taken back to the lab for the analysis preparation.

### Laboratory analysis

In the laboratory, the moist soil samples were crushed to pass through 2 mm sieve, and removed the roots and other debris by tweezers. The sieved soil samples were divided into two sub-samples for air-dried and stored at low temperature, respectively. A part of air-dried samples was sieved through 0.18 mm screen to measure the soil SOC and labile organic carbon (LOC). The moist samples about 200 g each sample were immediately stored at 4 °C to measure the soil microbial biomass carbon (MBC). SWC was determined by oven-dried at 105 °C to constant weight (approximately 24 h). BD was calculated as the ratio of dry soil weight by oven-dried at 105 °C for 24 h of the soil (volume: 100 cm^3^). Total soil porosity (P_t_), Eq. (1) was obtained from measured BD and soil particle density (2.65 g cm^−3^)^57^, the calculation equation according to the following:

1 $${P}_{t}=\left(1-\frac{{\rho }_{b}}{{\rho }_{p}}\right)\times 100\%$$

where P_t_ is the total soil porosity, *ρ*_b_ refers to the soil bulk density, and *ρ*_p_ refers to the soil density (2.65 g cm^−3^).

The pipette method was used to measure the soil particle size with Na hexametaphosphate after soil organic matter oxidation with H_2_O_2_ by a Laser Grain-size Analyzer (Mastersizer 3000, Malvern Instruments Inc., Worcestershire, UK) with international classification^58^, then calculated the proportions of the clay (< 0.002 mm), silt (0.002–0.02 mm), and sand (> 0.02 mm) contents.

The soil aggregates were measured by the dry-sieving method and wet-sieving method using soil aggregate analyzer (TTF-100, Shunlong experimental instrument factory, Shangyu city, China). A set of five stacking sieves with openings of 5, 2, 1, 0.5 and 0.25 mm were selected to determine the dry-stable aggregates and wet-stable aggregates with air-dried soil samples of 100 g and 50 g with three replicates respectively. The aggregates were divided into aggregates sized > 5 mm, 2–5 mm, 1–2 mm, 0.5–1 mm, 0.25–0.5 mm. The contents of > 0.25 mm mechanically stable aggregates (*R*_*0.25*_) were calculated using Eq. (2), for which the > 0.25 mm fraction was the most susceptible to changes in land use or management^40,59^, and soil aggregate fractions obtained by the dry-sieving method have been successfully used to analyze SOC pool^60^. The soil structural stability was characterized using the mean weight diameter (MWD), geometric mean diameter (GMD) of soil aggregates according to Eqs. (3) and (4)^61,62^. The > 0.25 mm percentage of aggregate disruption (PAD_0.25_) was calculated using Eq. (5).

2 $${R}_{0.25}=\frac{{\mathrm{M}}_{\mathrm{r}>0.25}}{{\mathrm{M}}_{\mathrm{T}}} \times 100\%$$

3 $$MWD\left(mm\right)={\sum }_{i=1}^{n}{X}_{i}{W}_{i}$$

4 $$GMD\left(mm\right)=Exp\left[\frac{\sum_{i=1}^{n}{W}_{i}\mathrm{ln}{X}_{i}}{\sum_{i=1}^{n}{W}_{i}}\right]$$

5 $${PAD}_{0.25}=\frac{({D}_{0.25} - {W}_{0.25})}{{\mathrm{D}}_{0.25}}\times 100\%$$

where *R*_*0.25*_ is the content of soil aggregates > 0.25 mm, *Mr*_>0.25_ is weight of aggregate > 0.25 mm, *M*_*T*_ is the total weight of soil tested. *X*_*i*_ is the mean diameter of each size classes (< 0.25 mm, 0.25–0.5 mm, 0.5–1 mm, 1–2 mm, 2–5 mm and > 5 mm), and *W*_*i*_ is the weight fraction of aggregates in size class *i*, and *n* is the number of size fractions. *D*_0.25_ is the > 0.25 mm dry-sieved aggregate content, and *W*_0.25_ is the > 0.25-mm water-stable aggregate content.

The total SOC concentrations were determined following the dry combustion method^63^ using a CHN analyzer. The MBC was measured by chloroform-fumigation extraction method^51,64^. LOC was determined by using 333 mmol L^−1^ KMnO_4_ Oxidation Method, and measured by the spectrophotometric of 565 nm wave length^65^. The total SOC was considered as equal to the total soil carbon because the measured inorganic carbon (carbonates) contents of the samples were almost nil^66^. Carbon Management Index (CMI) was calculated using the procedure outlined below ^65,66^, using the no-reclamation forest soil as reference sample:

6 $$\mathrm{Non}-\mathrm{labile }\,\,\mathrm{carbon }\left(\mathrm{NL}\right)=\mathrm{TOC}-\mathrm{LOC}$$

7 $$\mathrm{Lability\;of\;C }\left(\mathrm{L}\right)=\frac{\mathrm{C\;in\;fraction\;oxidized\;by\;KMnO}4}{\mathrm{C\;remaining\;unoxidized\;by\;KMnO}4 }=\frac{{C}_{L}}{{C}_{NL}}$$

8 $$\mathrm{Lability \; Index}\,\left(\mathrm{LI}\right)=\frac{\mathrm{Lability \; of \; C \; in \; sample \; soil}}{\mathrm{Lability \; of \; C \; in \; references \; soil }}$$

9 $$\mathrm{Carbon \; Pool \; Index }\left(\mathrm{CPI}\right)=\frac{\mathrm{Sample \; total \; C}}{\mathrm{Reference \; total \; C }}=\frac{{C}_{T}\;\mathrm{ Sample}}{{C}_{T}\;\mathrm{ Reference}}$$

10 $$\mathrm{Carbon\;Management\;Index }\;(\mathrm{CMI}) =\mathrm{CPI}\times \mathrm{LI}\times 100$$

### Statistical analysis

One-way ANOVA was carried out using the SPSS software, ver. 16.0 (IBM, USA) to analyze the differences of soil physical properties and soil carbon among different land use types. Means of the main effect were compared using Duncan multiple-range procedure test at *P* ≤ 0.05 for significance. Pearson correlation coefficients were used for the correlation analysis between soil physical properties and soil carbon. All the figures were produced using Origin 10.0 (Originlab, Northampton, Massachusetts, USA).

## Supplementary information

Supplementary Information
