# Supplementary material for: Variation of soil organic carbon and physical properties in relation to land uses in the Yellow River Delta, China
Source: Sci Rep. 2020 Nov 23;10:20317. doi: 10.1038/s41598-020-77303-8 (PMC7683548; doi:10.1038/s41598-020-77303-8)
Supplement: Supplementary file 1 — Supplementary Information [file 41598_2020_77303_MOESM1_ESM.docx]

Variation of soil organic carbon and physical properties in relation to land uses in the Yellow River Delta, China

Shuying Jiao^1^, Junran Li^2^, Yongqiang Li^1^*, Ziyun Xu^1^, Baishu Kong^1^, Ye Li^1^ & Yuwen Shen^3^

^1^ College of Resources and Environment, National Engineering Laboratory for Efficient Utilization of Soil and Fertilizer Resources, Shandong Agricultural University, No. 61 Daizong Street, Tai’an, Shandong 271018, China

^2^ Department of Geosciences, The University of Tulsa, Tulsa, OK 74104, USA

^3^ Shandong Academy of Agricultural Sciences, Institute of Agricultural Resources and Environment, No. 202 Gongyebei Road, Jinan, Shandong 250100, China

Correspondence and requests for materials should be addressed to Y. L. (email: yongqiangli@sdau.edu.cn)

| **Soil depths** | **land uses** | **>5mm** | **5-2mm** | **2-1mm** | **0.5-1mm** | **0.5-0.25mm** | ***R_0.25_*** |
| --- | --- | --- | --- | --- | --- | --- | --- |
| 0-5 cm | AL | 0 | 0±0b | 0.23±0.12b | 0.95±0.32b | 0.01±0.01b | 1.19±0.38b |
|  | GL | 0 | 0.73±0.47a | 0.94±0.34a | 1.81±0.47a | 0.02±0.01a | 3.51±1.05a |
|  | FL | 0 | 0.04±0.01b | 0.06±0.03b | 0.18±0.15c | 0±0c | 0.29±0.14c |
| 5-10 cm | AL | 0 | 0.28±0.10a | 0.22±0.08a | 0.75±0.18a | 0.02±0.01a | 1.26±0.31a |
|  | GL | 0 | 0.38±0.11a | 0.32±0.14a | 0.61±0.21a | 0.02±0.01a | 1.32±0.30a |
|  | FL | 0 | 0±0b | 0.04±0.02b | 0.07±0.03b | 0±0b | 0.11±0.05b |
| 10-20 cm | AL | 0 | 0.21±0.02a | 0.22±0.10a | 0.56±0.14b | 0.01±0b | 1.00±0.25b |
|  | GL | 0 | 0.22±0.08a | 0.28±0.09a | 1.05±0.46a | 0.03±0.01a | 1.57±0.50a |
|  | FL | 0 | 0±0b | 0.08±0.05b | 0.10±0.04b | 0±0c | 0.19±0.05c |
| 20-30 cm | AL | 0 | 0±0b | 0.08±0.04a | 0.32±0.19b | 0.01±0b | 0.41±0.21b |
|  | GL | 0 | 0.13±0.05a | 0.44±0.61a | 2.41±2.24a | 0.05±0.02a | 3.04±2.87a |
|  | FL | 0 | 0±0b | 0.06±0.01a | 0.04±0.03b | 0±0b | 0.11±0.04b |
| 30-50 cm | AL | 0 | 0±0b | 0.02±0.04b | 0.10±0.10b | 0±0b | 0.12±0.14b |
|  | GL | 0 | 0.10±0.05a | 0.35±0.24a | 1.29±0.74a | 0.03±0.02a | 1.77±1.00a |
|  | FL | 0 | 0.02±0.04b | 0.17±0.17ab | 0.53±0.56b | 0±0b | 0.71±0.71b |

**Table S1**. Effect of land uses on soil water-stable aggregates (%) at 0-50 cm depth (mean±SD). Different letters with the same soil depth indicate significant difference (n=5, p<0.05) between different land uses by one-way ANOVA. *R_0.25_* is aggregates of diameter > 0.25 mm.
